# The 2d No of the Register

**Published:** 1848-01

**Authors:** 


### THE 2D NO. OF THE REGISTER.

We present to our subscribers the 2d No. of the Register some two or three weeks later than we had intended. Several causes have operated to keep back the present issue. During almost the entire months of December and January we were confined to our bed and room by a severe attack of fever. Several of our contributors knowing this, deferred sending in their contributions until the last of the month. We wish it to be remembered that contributions to the Register should be sent in by the 1st of the months of January, April, July and October. These are the months for the issuing of the Register, and we should be glad to have it from press in time for distribution by the middle of the month. The printers agree to do *their* part of the work in ten days, yet delays often occur in printing offices, and it is better to provide against them.

There are many of our old practitioners throughout the west, that we know could give much instructive matter for the pages of the Register, who have not yet "thrown in their mite." As yet, nearly if not quite half our contributors are graduates of the "Ohio College of Dental Surgeons." It affords us much gratification to know they have not lost their taste for study, and we refer to this to show what we may expect to be the effect of such institutions. We know that many, who are from experience best qualified to write, are so occupied in their profession that they have but little time to devote in that way; yet this should not entirely excuse any one.—CIN. ED.

